# Macro-Encapsulation of Inorganic Phase-Change Materials (PCM) in Metal Capsules

**DOI:** 10.3390/ma11091752

**Published:** 2018-09-17

**Authors:** Stephan Höhlein, Andreas König-Haagen, Dieter Brüggemann

**Affiliations:** Chair of Engineering Thermodynamics and Transport Processes (LTTT), Center of Energy Technology (ZET), University of Bayreuth, Universitätsstraße 30, 95440 Bayreuth, Germany; Andreas.Koenig-Haagen@uni-bayreuth.de (A.K.-H.); Dieter.Brueggemann@uni-bayreuth.de (D.B.)

**Keywords:** phase-change material, macro-encapsulation, thermal energy storage, latent heat storage, salt hydrate, waste heat, corrosion

## Abstract

The design of phase-change material (PCM)-based thermal energy storage (TES) systems is challenging since a lot of PCMs have low thermal conductivities and a considerable volume change during phase-change. The low thermal conductivity restricts energy transport due to the increasing thermal resistance of the progressing phase boundary and hence large heat transfer areas or temperature differences are required to achieve sufficient storage power. An additional volume has to be considered in the storage system to compensate for volume change. Macro-encapsulation of the PCM is one method to overcome these drawbacks. When designed as stiff containers with an air cushion, the macro-capsules compensate for volume change of the PCM which facilitates the design of PCM storage systems. The capsule walls provide a large surface for heat transfer and the thermal resistance is reduced due to the limited thickness of the capsules. Although the principles and advantages of macro-encapsulation have been well known for many years, no detailed analysis of the whole encapsulation process has been published yet. Therefore, this research proposes a detailed development strategy for the whole encapsulation process. Various possibilities for corrosion protection, fill and seal strategies and capsule geometries are studied. The proposed workflow is applied for the encapsulation of the salt hydrate magnesiumchloride hexahydrate (MCHH, MgCl${}_{2}\;\cdot \;6$H${}_{2}$O) within metal capsules but can also be assigned to other material combinations.

## 1. Introduction

Phase-change materials (PCMs) possess the ability to store large amounts of thermal energy when applied around their phase-change temperature. This enables the design of compact thermal energy storage systems (TES). As drawbacks, non-metallic PCMs have comparatively low thermal conductivities and a considerable volume expansion due to the phase-change [1]. Furthermore, interactions between the PCM and the TES container have to be considered, especially when combining inorganic PCMs and metallic materials [2,3,4,5]. Encapsulation has been realised for a lot of different PCMs, application temperatures and capsule materials to overcome the mentioned challenges [6,7,8]. Commercial solutions are already available for the low and medium temperature sector like the sphere shaped nodules from cristopia [9] or flat plates and cylinders from PCM Products [10] which are made of polymers. Milián et al. [6] have proposed a classification of the available encapsulation methods based on the shape of the material, as core-shell and shape-stabilized PCMs. The core-shell methods have been further classified as chemical, physicochemical and mechanical packaging, whereby the latter one describes the encapsulation method investigated in this paper. Furthermore, this research focuses on the encapsulation of PCM for a mobilized TES application and a temperature range between 90 and 150 ${}^{\circ }C$. The inorganic PCM magnesiumchloride hexahydrate (MCHH, MgCl${}_{2}\;\cdot \;6$H${}_{2}$O) is utilized as storage material for the desired application, since other candidates like the sugar alcohols xylitol and erythritol suffer from supercooling [11]. Metals are considered as capsule materials due to their temperature resistance, non-flammability, mechanical strength and high thermal conductivity.

There are several studies concerning the encapsulation of inorganic PCM within metal capsules. Cylindrical steel capsules, filled with sodium nitrate (NaNO${}_{3}$) and an eutectic of sodium chloride and magnesium chloride (NaCl-MgCl${}_{2}$, 57% mole fraction NaCl) have been investigated by Zheng et al. [12]. They have decided to apply cylinders after experimenting with various, not further defined, geometries. Finite element models have been utilized to examine adequate capsule strength and transient heat transfer characteristics with the result that the characteristic dimension of the cylinder should be less than 10 cm [12]. Zhang et al. [13] have encapsulated a NaNO${}_{3}$-KNO${}_{3}$ eutectic PCM mixture within stainless steel (AISI 321) cylinders. They have applied COMSOL Multiphysics [14] to consider the alternating pressure due to phase-change inside the capsule to ensure mechanical stability of the encapsulation. However, no information about the amount of PCM within the capsule is given [13]. Numerical simulations to investigate the stresses in spherical and cylindrical capsules have been performed by Blaney et al. [15]. The materials for the sphere and the cylinder have been assumed as nickel and stainless steel (316L) respectively, and the PCM was zinc. The pressure alternation due to phase-change with different amounts of PCM within the capsule has been calculated applying the combined gas law neglecting thermal expansion of the encapsulation material. The cylinder has been identified as the most viable geometry for encapsulation [15]. Lane [16] has used steel aerosol cans to encapsulate Mg(NO${}_{3}$)${}_{2}\;\cdot \;$6H${}_{2}$O. The cans have been filled with the liquid PCM, cooled down to the solid state, blanked with nitrogen and finally sealed. Tin-plated food cans have been applied to encapsulate MCHH by Gonçalves and Probert [17]. An additional lacquer layer has been applied for corrosion protection. Ibáñez et al. [18] have encapsulated a mixture of sodium acetate and graphite in commercial aluminium bottles.

None of the reviewed research articles give a detailed description of the whole encapsulation process itself. A similar conclusion has been drawn by Jacob and Bruno [8] who emphasized the need for further technical investigations on shell composition for encapsulation. Therefore, this research proposes a design strategy with detailed consideration of the whole encapsulation process, starting from the boundary conditions given by the TES application to the material selection and the encapsulation process. The design strategy is exemplarily applied to the PCM MCHH and metals as encapsulation shell. Nevertheless, the principle is applicable to other materials as well like organic PCMs in metal or plastic containers.

## 2. Analysis of the Development Process of Macro-Encapsulated TES

**Figure 1:**
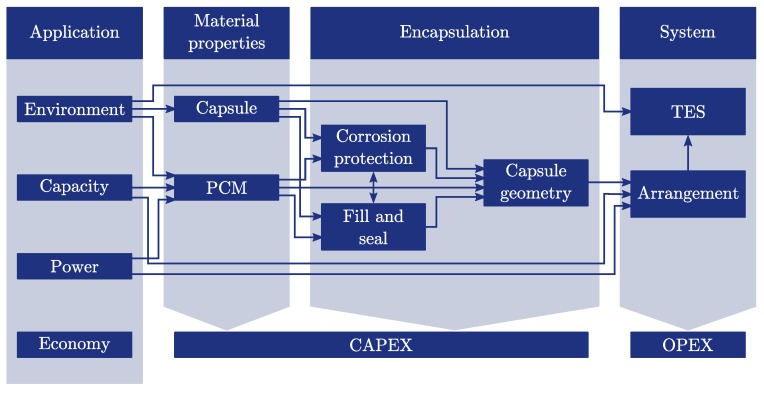
Workflow for the development of a macro-encapsulated thermal energy storage system consisting of metal capsules. The interconnections are described in detail in the corresponding sections.

This section describes the novel strategy for the development of macro-encapsulated TES. The strategy is based on the analysis of various publications in the field of macro-encapsulation and the experience of the authors gained in different encapsulation projects. The flowchart of the proposed workflow is depicted in Figure 1. The process is divided into four main levels: application, material properties, encapsulation and system. Each level and its set of parameters as well as the influences among each other are described in the following sections with special focus on the encapsulation process.

### 2.1. Application

The environment (Figure 1) determines the boundary conditions under which the macro-encapsulated TES is operated. Boundary conditions describe if it is a stationary or mobile application, the available heat transfer fluid and its mass flow rates, the acceptable space for the storage system, safety requirements (flammability/hazardous materials), operating temperatures and so on. The capacity and the power are the required storage capacity and power of the TES application and the economy describes the acceptable capital expenditures (CAPEX) and operational expenditures (OPEX). The economy is influenced by all the steps, but in the interest of clarity it is not connected to the different fields. Examples for different applications and their boundary conditions may be found in the book of Hauer et al. [19] and the review article of Miró et al. [20].

### 2.2. Material Properties

The choice of the capsule (Figure 1) material is affected by the environmental conditions of the application; for example, the operating temperatures may specify if plastics or metals can be applied. Another criterion for the preselection may be the imposed safety requirements; for example, to avoid flammable materials within the TES. The material properties, like yield strength, density, heat capacity, thermal conductivity and so on of the selected capsule materials have to be known.

The choice of the PCM (Figure 1) is affected by the environment, the required capacity and power of the application. The influence of the environment parameters is similar to the discussion above. The melting and solidification temperature of the PCM and possibly safety requirements are usually the key parameters for the preselection of materials. The solidification temperature can be affected by supercooling of the PCM which is a well known phenomena and may restrict the applicability of the PCM. One way to suppress or decrease supercooling is the addition of nucleating agents to the PCM. Examples for suitable material combinations are summarized in the research of Lane [21] and the review article of Xie et al. [22]. Other methods like seeding with crystals from the same material [23], electrical nucleation [24] or ultrasonic irradiation [25] are possible as well but difficult to apply to macro-encapsulated PCM. Since the degree of supercooling depends on the PCM volume investigations with sample sizes close to macro-capsule scale are recommended. It may be advantageous to choose PCM with high volumetric storage densities to meet the capacity requirements of the application. In contrast, a high thermal conductivity could be the crucial property to reach the power requirements. In any case, the thermophysical properties like melting enthalpies and temperatures, heat capacities, densities and thermal conductivities have to be known to design the storage system. When salt hydrates are applied as PCMs the phase-change can occur with different phase equilibrium behaviours. Lane [26] has categorized congruent-melting, quasi-congruent-melting, congruent-melting isomorphous and eutectic materials as well-behaved materials since they indicate a good stability for practical applications. In contrast semicongruent-melting and incongruent-melting PCMs have been defined as difficult materials because these materials can suffer from segregation which requires the deployment of additional thickeners, gellants or stirring of the PCM [26]. The long-term stability of the properties over an appropriate number of melting and solidification cycles has to be examined as well. Methods for testing the stability and some results for various PCMs are described in the review of Rathod and Banerjee [27].

### 2.3. Encapsulation

#### 2.3.1. Corrosion Protection

**Figure 2:**
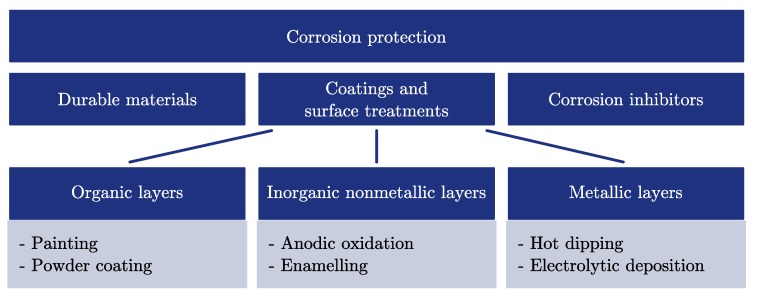
Summary of different corrosion protection methods which can be applied to macro-encapsulated phase-change materials.

The corrosion protection (Figure 1) has to be compatible with the chosen PCM and the capsule. This step is specific for the encapsulation within metal containers focused on in this study. Nevertheless, capsules made of plastic may suffer from compatibility problems as well [28,29]. Some types of coatings or surface treatments are only applicable to certain materials; for example, the anodic oxidation with materials which can build oxide layers. Coating adhesion and the durability of the coating against temperature-induced expansion of the capsule material are further topics of interest. In general, there are three methods to deal with corrosion (Figure 2).

##### Durable Materials

**Figure 3:**
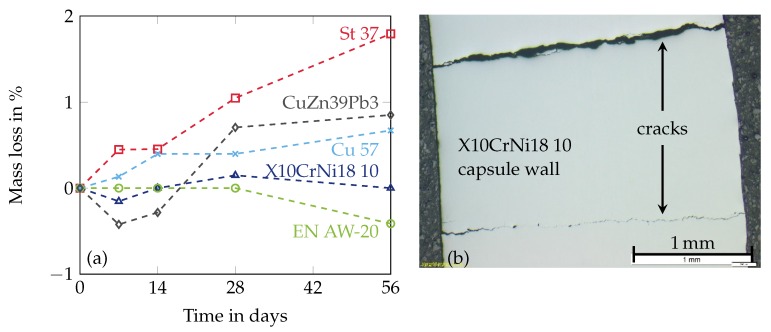
Results of the corrosion experiments with MCHH and various metals at a temperature of 122 ${}^{\circ }C$ (**a**) and scanning electron microscope recording of a capsule made of stainless steel (**b**). Reproduced with permission from Brüggemann et al. [30].

One way is to find durable encapsulation materials for the desired PCM. The combination of MCHH applied with different metals has been investigated by some research groups. El-Sebaii et al. [5] have performed corrosion experiments with stainless steel and aluminium in contact with MCHH imposed to 500 melting and solidification cycles. Both metals do not seem to be resistant to corrosion in the present form but there is no detailed information about the applied alloys. Ushak et al. [3] have investigated the corrosion behaviour of stainless steel (316L), aluminium (alloy 1100) and copper (C11000) in contact with MCHH up to 1500 h at 120 ${}^{\circ }C$. In contrast to the studies of El-Sebaii et al. [5] the metal samples have not been completely submerged in the PCM to get information about the behaviour at the liquid-gas interface. Neither stainless steel nor aluminium or copper seem suitable as container materials. In a previous study the authors have investigated mild steel (St 37), stainless steel (X10CrNi18 10), aluminium (EN AW-20), copper (Cu 57) and brass (CuZn39Pb3) in contact with MCHH [30]. Small plates of each material with the dimensions 30 mm × 15 mm × 2 mm have been submerged within the molten MCHH at a temperature of 122 ${}^{\circ }C$. The determined mass losses after 7, 14, 28 and 56 days are visualized in Figure 3a. The aluminium alloy and stainless steel have indicated good compatibility with MCHH in the corrosion experiments. Nevertheless, capsules made of pure stainless steel and aluminium without a specific corrosion protection have failed later on due to crack and pitting corrosion (Figure 3b) [30].

##### Coatings and Surface Treatments

Coatings and surface treatments can be subdivided into organic, inorganic nonmetallic and metallic layers [31] (Figure 2). This kind of corrosion protection allows the application of comparatively cheap base materials; for example, mild steel instead of stainless-steel. Nevertheless, additional costs are caused by the coating or surface treatment itself. Gonçalves and Probert [17] have investigated a storage unit operated with MCCH encapsulated in food cans which have been coated with a lacquer coating to prevent corrosion. Hale et al. [32] have suggested oxide coatings for aluminium as extremely effective for most chemicals. Oxide layers, generated by anodisation of the aluminium, enhance the natural oxide layer thickness of aluminium from 1–3 nm up to 150 μm and are insoluble in the pH range from 4.5 to 8.5 [33]. The anodisation process requires good accessibility to the inner surface of the capsule and a continuous exchange of the electrolyte [34]. Oxide layers are conversion layers which are formed from the base material itself. Therefore, only approximately 1/3 of the oxide layer thickness is added to the base material and influences its outer dimensions [35]. In contrast, powder coatings add an additional layer of a thickness between 40 and 120 μm [31] to the base material which has to be considered when different components of a capsule have to fit together (e.g., the capsule and the closure). Furthermore, the coating may have a considerably smaller thermal conductivity than the base material and can act as an undesired insulation layer. Typical values for the thermal conductivity of the oxide layer and powder coatings are 20 to 30 W/(mK) [33] and 0.15 to 0.5 W/(mK) [31,36], respectively.

##### Corrosion Inhibitors

There is only limited information about the application of corrosion inhibitors in PCMs in general and no literature for MCHH in particular. Lane [26] has explained the rare deployment of inhibitors by the possible interference of the phase-change characteristics of the PCM. Hale et al. [32] have listed some components for acids, alkaline compounds and water in contact with aluminium. Zhang et al. [37] have tested methionine and proline as inhibitors in inorganic PCM solutions in contact with 1045 carbon steel and have reached a maximal protection efficiency up to 94%.

#### 2.3.2. Fill and Seal

**Figure 4:**
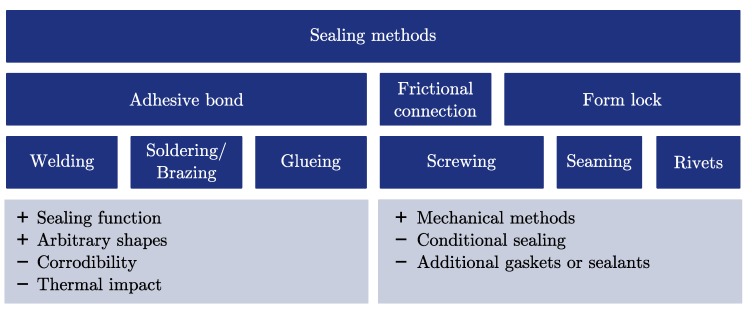
Summary of different sealing methods which can be applied to macro-encapsulated phase-change materials.

The chosen PCM determines the fill and seal (Figure 1) process of the capsule; for example, as a solid block or powder or in its liquid state. A collection of various sealing methods is depicted in Figure 4. The methods are categorised in adhesive bondings, frictional connections and form lock methods. The chosen capsule material influences the possibilities of sealing the capsule; for example, if it is feasible to apply welding, soldering or adhesive bonding for the sealing process. It should be considered that the corrosion protection and the fill and seal process have to be compatible with each other. Welding or soldering may require high temperatures above the decomposition or ignition temperature of the PCM and the corrosion protection layer.

There are few studies about sealing methods for inorganics within metal capsules. Zheng et al. [12] have sealed steel capsules filled with the PCMs NaNO${}_{3}$ or NaCl–MgCl${}_{2}$ by welded end caps. Fleischer [38] has proposed that containers should be permanently sealed by welding, soldering or brazing, since high temperature gaskets and sealants may suffer from leakage. Dong et al. [39] have applied rivets to close the filling opening of spherical capsules. Since standard rivets are not gas-tight, an additional epoxy resin has been applied for sealing [39].

#### 2.3.3. Capsule Geometry

The desired capsule geometry (Figure 1) is restricted by the capsule material which defines how it can be processed; for example, if it is possible to produce a desired geometry by forming processes. The selected corrosion protection method has to be applicable to the capsule geometry. Some methods like the anodic oxidation or powder coating, may need adequate access to the inner surface of the capsule, which can be problematic when utilizing spheres with a small filling hole or in general geometries with small inner diameters. For PCMs which suffer from phase segregation, it may be advantageous to design flat geometries to limit the possible segregation length. The way how the capsule is filled and sealed influences the pressure within the capsule and hence the required wall thickness.

### 2.4. System

The arrangement (Figure 1) determines the way how the capsules are aligned within the storage system. They can be applied as structured or unstructured packing which results in different storage densities, heat transfer coefficients and surfaces. Hence, the adjustment of the arrangement of the capsules can be used to meet the required capacity and power requirements. Finally, the TES will be equipped with the arranged capsule packing and operated with the environment conditions given by the application.

## 3. Exemplary Implementation

The encapsulation concept presented in the previous chapter (Figure 1) is now applied to a waste heat storage application. The environment conditions may require non flammable and non hazardous materials and an operating temperature in the range between 90 and 150 ${}^{\circ }C$. There are different salt hydrates and sugar alcohols available as PCMs which meet these requirements. The characterization of potential PCMs and the final choice of MCHH as storage material has been described in a previous paper [11] and some of the most important properties are tabulated in Table 1.

**Figure 5:**
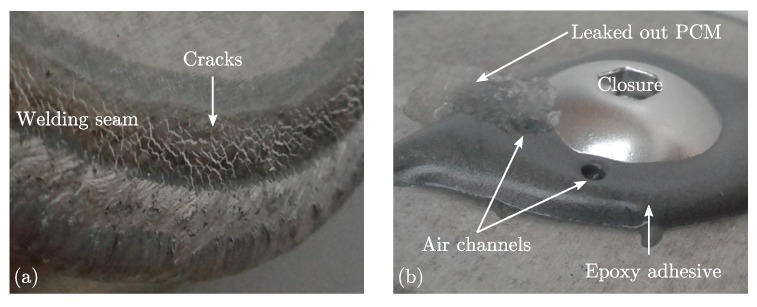
Summary of failed sealing methods for aluminium capsules. (**a**) Welded closure; (**b**) Glued closure.

Compatibility investigations between the MCHH and different metal capsule materials have revealed that none of the metals is resistant at its pure form [30]. Therefore, aluminium (alloy: EN AW-6060 [40]) was chosen as base material for the capsules since it is cheap, of low weight and easy to process. An additional anodized layer was applied to meet the required corrosion resistance. The best way of filling the MCHH into the capsules was in its liquid state, since filling in form of powder or flakes results in low degrees of filling due to the porosity. The melting of the PCM had been conducted in a closed container to prevent loss of crystal water of the MCHH and afterwards it was filled into the capsule. Mechanical methods were applied for the sealing process because the thermal impact of methods like welding or soldering could destroy the corrosion protection layer as well as the PCM. A failed example of a welded capsule is depicted in Figure 5a. The figure shows an enlarged section of the welding seam of an aluminium capsule. The capsule had been filled with the PCM and afterwards the end cap was welded. The high energy input of the laser welding system had induced a strong temperature rise within the heat-affected zone and the MCHH in the neighbourhood started to foam. The foam had penetrated the liquid welding seam causing visible white cracks in the cooled down seam which leaked later on. An example of a closure which was sealed by glueing is depicted in Figure 5b. The applied epoxy adhesive (Loctite EA 9514 [41]) was heat cured at temperatures above the melting point of MCHH. The expansion of the PCM and the air within the capsule during curing had induced air channels in the bond which leaked later on.

Due to the aforementioned drawbacks, the following mechanical methods were applied to seal the capsules. A gasket made of FKM (fluoroelastomer) was applied as sealing element between the PCM and the ambient as this material is compatible with the selected materials and is stable up to the desired temperatures of 150 ${}^{\circ }C$ [42]. Screws were utilized to fix the end caps at the desired position.

**Figure 6:**
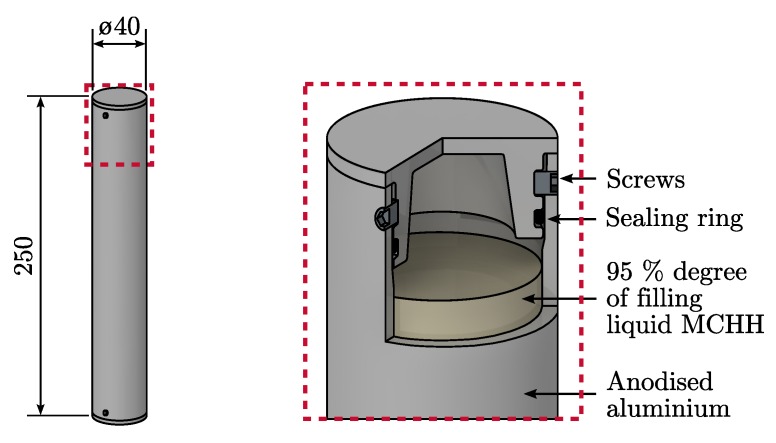
Final encapsulation concept, consisting of seamless aluminium pipes as base material.

Seamless aluminium pipes were selected as base materials for the capsules, since they show good accessibility for the chosen corrosion protection and applicability of the sealing concept. A detailed view of the final encapsulation concept is depicted in Figure 6. The pipes have a wall thickness of 3 mm, an outer diameter of 40 mm and the overall capsule length is 250 mm. The PCM occupies in its liquid state 95% of the capsule volume which corresponds to a volume of 0.194 ${\mathrm{dm}}^{3}$. The total mass of the PCM capsule is 554 g, whereby 281 g are from the PCM. A single macro-capsule can store 47 kJ of thermal energy when latent heat is considered as the only energy storage mechanism and sensible heat of the capsule shell and the PCM is neglected. Referred to the capsule outer volume the storage density is 150 kJ/dm${}^{3}$.

The proposed cylindrical macro-capsules can be arranged in a storage system in various ways resulting in different storage powers and capacities. The highest storage capacities of cylinders of equal diameters can be achieved with uniform packings by applying a triangular alignment with a packing density of up to 90%. This type of capsule packing would result in a latent heat storage capacity of 135 MJ per cubic meter storage container volume. The storage power is also influenced by the arrangement of the capsules since the alignment has an impact on the flow conditions and, hence, the heat transfer within the storage system. There are a lot of other factors discussed in Section 2.1 which influence the storage power, too. In any case, a careful consideration of heat transfer, storage capacity and pressure drop is necessary to build up storage systems based on the proposed macro-capsules.

From an economic point of view, the proposed encapsulation concept is very expensive. As depicted in Table 2, the specific costs of 93.19 €/dm${}^{3}$ encapsulated PCM for the test capsules are caused to 48% by the manufacturing process. The transition from individual to mass fabrication may decrease the encapsulation cost considerably. The chosen corrosion protection and the capsule material are responsible for 26% and 25% of the total costs, respectively. Higher quantities and capsules with decreased wall thickness may lower their proportion. The PCM itself is responsible for 1% of the total costs. Expenses for the filling and sealing of the capsule cannot be quantified.

Cost effective alternatives for the capsule shell can be products which are available as mass products on the market. Food cans are very cheap, but, in the available configuration, are not compatible with the PCM MCHH at elevated temperatures. Experiments with food cans revealed a decomposition of the lacquer layer and later corrosion and leakage of the shell. Typical cost of about 0.3 € for a food can with an inner volume of 0.425 $d^{3}$m [43] and a degree of filling of 95% would result in specific cost of 0.74 €/dm${}^{3}$ encapsulated PCM.

## 4. Conclusions

A development strategy for macro-encapsulation of PCM was presented. The procedure was discussed in detail with the focus on encapsulation of inorganic PCMs within metal capsules. The interactions between different process steps were discussed and examples were given. The analysis reveals that the final geometry of a macro-capsule is not necessarily a parameter of choice but a result of various upstream process steps.

The proposed workflow was exemplarily applied to the encapsulation of the inorganic salt hydrate magnesiumchloride hexahydrate within metal capsules. The resulting macro-capsules consist of anodized aluminium pipes which are hermetically sealed with mechanical methods. From an economic point of view, the developed macro-capsules are still very expensive, which is mainly caused by the individual fabrication. Low cost encapsulation concepts applying food or aluminium cans manufactured in mass production are proposed as a cost-efficient alternative for future work.

## Figures and Tables

**Table 1:** Thermophysical properties of the applied PCM MCHH (adapted from [11]).

Property	Value
Melting Temperature	115.1 ± 0.1 ${}^{\circ }C$
Supercooling *	2.8 K
Melting enthalpy between 114–118 ${}^{\circ }C$	166.9 ± 1.2 J/g
Solid state heat capacity at 100 ${}^{\circ }C$	1.83 ± 0.06 J/(gK)
Liquid state heat capacity at 120 ${}^{\circ }C$	2.57 ± 0.06 J/(gK)
Solid state density at 20 ${}^{\circ }C$	1.5955 ± 0.0002 $g/{\mathrm{cm}}^{3}$
Liquid state density at 120 ${}^{\circ }C$	1.4557 ± 0.0004 $g/{\mathrm{cm}}^{3}$
Solid state thermal conductivity at 110 ${}^{\circ }C$	0.70 ± 0.05 W/(mK)
Liquid state thermal conductivity at 120 ${}^{\circ }C$	0.63 ± 0.04 W/(mK)

* Sample size 100 g.

**Table 2:** Estimated costs for the proposed macro-capsules. The costs for manufacturing, capsule material and corrosion protection are based on the invoices for the production and the costs of the PCM are taken from a previous publication [11].

Description	Costs Per Capsule in €/capsule	Costs Per $dm^{3}$ PCM in €$/dm^{3}$	Share in %
Manufacturing	8.68	44.64	48
Capsule material	4.55	23.38	25
Corrosion protection	4.68	24.05	26
PCM (MCHH)	0.22	1.11	1
Total	18.11	93.19	100

## Abbreviations

The following abbreviations are used in this manuscript:

CAPEX	Capital expenditures
MCHH	Magnesiumchloride hexahydrate
OPEX	Operational expenditures
PCM	phase-change material
TES	Thermal energy storage
